# 1-(4-Fluoro­benz­yl)-2-(pyridin-2-yl)-1*H*-benzimidazole

**DOI:** 10.1107/S1600536814005947

**Published:** 2014-03-26

**Authors:** Ömer Çelik, Fırat Anĝay, Mustafa Gündoĝan, Mahmut Ulusoy

**Affiliations:** a Department of Physics, Faculty of Education, Dicle University, 21280, Diyarbakır, Turkey; b Department of Physics, Institute of Sciences, Dicle University, 21280, Diyarbakır, Turkey; c Department of Chemistry, Faculty of Science & Art, Harran University, 63300, Şanlıurfa, Turkey

## Abstract

In the title compound, C_19_H_14_FN_3_, the dihedral angles between the benzimidazole unit (r.m.s. deviation= 0.017 Å) and the pyridine and benzene rings are 24.46 (4) and 81.87 (3)°, respectively. In the crystal, mol­ecules are stacked along the *a*-axis direction by C—H⋯π inter­actions.

## Related literature   

For the use of 2-(2-pyrid­yl)benzimidazole in coordination chemistry, see: Boca *et al.* (1997 ▶); De Castro *et al.* (1991 ▶); Khalil *et al.* (2001 ▶); Maekawa *et al.* (1994 ▶). For deprotonation of the NH group in 2-(2-pyrid­yl)benzimidazole, see: Chiswell *et al.* (1964 ▶); Harkins *et al.* (1956 ▶); Haga (1983 ▶). For functionalization of 2-(2-pyrid­yl)benzimidazole, see: Ali *et al.* (1998 ▶); Hossain *et al.* (2001 ▶); Sahin *et al.* (2010 ▶). For related structures, see: Çelik *et al.* (2007 ▶, 2009 ▶).
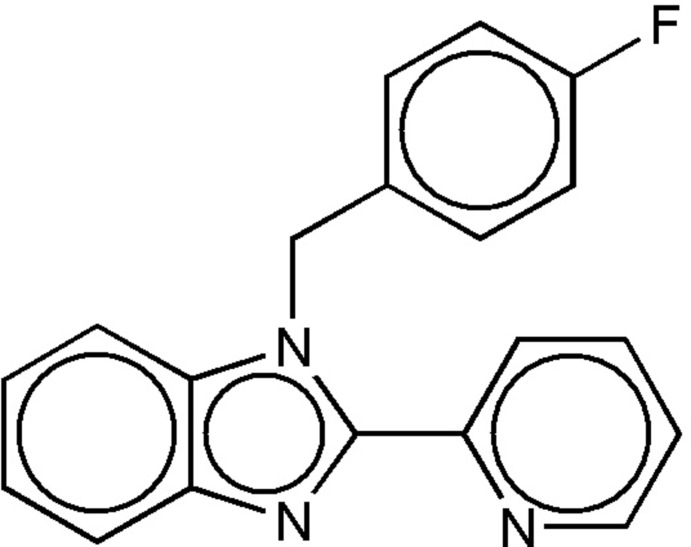


## Experimental   

### 

#### Crystal data  

C_19_H_14_FN_3_
*M* *_r_* = 303.33
Monoclinic, 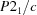

*a* = 4.7363 (5) Å
*b* = 15.4102 (17) Å
*c* = 20.953 (2) Å
β = 95.363 (8)°
*V* = 1522.6 (3) Å^3^
*Z* = 4
Mo *K*α radiation
μ = 0.09 mm^−1^
*T* = 296 K
0.2 × 0.2 × 0.2 mm

#### Data collection  

Bruker APEXII CCD diffractometer
Absorption correction: multi-scan (Blessing, 1995 ▶) *T*_min_ = 0.984, *T*_max_ = 0.984
13325 measured reflections
3133 independent reflections
2355 reflections with *I* > 2σ(*I*)
*R*_int_ = 0.027

#### Refinement  

*R*[*F* ^2^ > 2σ(*F* ^2^)] = 0.038
*wR*(*F* ^2^) = 0.103
*S* = 0.93
3133 reflections
208 parameters
1 restraint
H-atom parameters constrained
Δρ_max_ = 0.14 e Å^−3^
Δρ_min_ = −0.17 e Å^−3^

### 

Data collection: *APEX2* (Bruker, 2007 ▶); cell refinement: *SAINT* (Bruker, 2007 ▶); data reduction: *SAINT*; program(s) used to solve structure: *SHELXS97* (Sheldrick, 2008 ▶); program(s) used to refine structure: *SHELXL97* (Sheldrick, 2008 ▶); molecular graphics: *ORTEP-3 for Windows* (Farrugia, 2012 ▶); software used to prepare material for publication: *WinGX* (Farrugia, 2012 ▶).

## Supplementary Material

Crystal structure: contains datablock(s) I, global. DOI: 10.1107/S1600536814005947/bq2393sup1.cif

Structure factors: contains datablock(s) I. DOI: 10.1107/S1600536814005947/bq2393Isup2.hkl

Supporting information file. DOI: 10.1107/S1600536814005947/bq2393Isup3.cml

CCDC reference: 992223

Additional supporting information:  crystallographic information; 3D view; checkCIF report

## Figures and Tables

**Table 1:** Hydrogen-bond geometry (Å, °) *Cg* is the centroid of the C1–C6 ring.

*D*—H⋯*A*	*D*—H	H⋯*A*	*D*⋯*A*	*D*—H⋯*A*
C13—H13*A*⋯*Cg* ^i^	0.97	2.94	3.486 (2)	117

Symmetry code: (i) 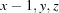.

**Fig. 1.**
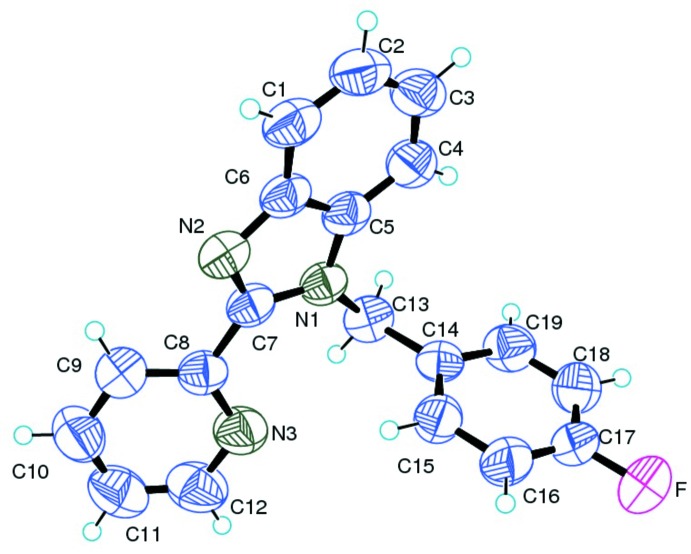
ORTEP III diagram of the compound, showing the molecular numbering scheme. Displacement ellipsoids are drawn at 50% probability for all atoms except H.

**Fig. 2.**
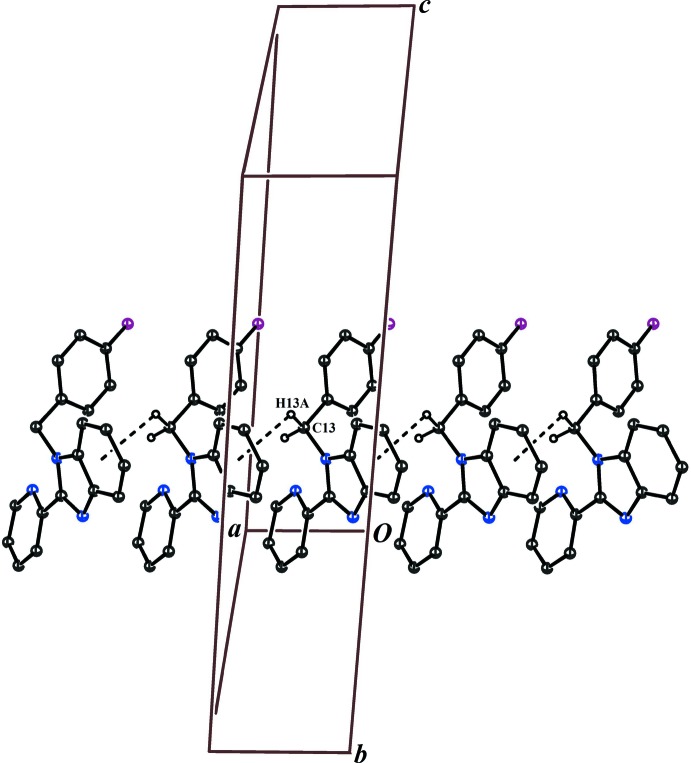
The stacking of the title compound along a-axis with C—H···π type hydrogen-bond interactions.

## supplementary crystallographic information

### 1. Comment

The N—N type ligand system, 2-(2-pyridyl)benzimidazole has a venerable history in coordination chemistry (Harkins *et al.*, 1956; Chiswell *et al.*, 1964; De Castro *et al.*, 1991; Maekawa *et al.*, 1994; Khalil *et al.*, 2001; Boca *et al.*, 1997). Many of the reported complexes of 2-(2-pyridyl) benzimidazole have been of interest because of the possibility of deprotonation of the NH group of the imidazole unit, converting the ligand from neutral to anionic form with different properties (Harkins *et al.*, 1956; Chiswell *et al.*, 1964; Haga, 1983). The functionalization of 2-(2-pyridyl)benzimidazole at the externally directed NH position allows simple incorporation of 2-(2-pyridyl)benzimidazole units (Sahin *et al.*, 2010; Hossain *et al.*, 2001; Ali *et al.*, 1998).

The molecular structure of title compound is shown in Figure 1. In the compound, the bond lengths of F—C17, N3—C12 and C1—C2 are 1.3671 (18) Å, 1.336 (2) Å and 1.373 (2) Å, respectively. C18—C17—F and N3—C8—C7 bond angles are 118.58 (13)° and 117.91 (13)°. F—C17—C18—C19 and C12—N3—C8—C7 torsion angles are -178.74 (15)° and -178.95 (14)°. Similar results are observed in the study of (Çelik *et al.*, 2009; Çelik *et al.*, 2007).

In the title compound, (Fig. 1), four planes were named as P1(N1/C5/C6/N2/C7), P2(N3/C8/C9/C10/C11/C12), P3(C1/C2/C3/C4/C5/C6), P4(C14/C15/C16/C17/C18/C19) and P5(N1/C5/C4/C3/C2/C1/C6/N2/C7). The five- and six-membered rings (N1/C5/C6/N2/C7) and (C1—C6) of the benzimidazole groups are almost co-planar with maximum deviations of -0.011Å for C5 and -0.017 Å for C6, respectively. Moreover the maximum deviations from P2 plane of C8, P4 plane of C17 and P5 plane of N2 are -0.007 Å, -0.003 Å and -0.034 Å, respectively.

In the compound, dihedral angles between P1—P2, P1—P3, P1—P4, P1—P5, P2—P3, P2—P4, P2—P5, P3—P4, P3—P5 and P4—P5 are 23.37 (5)°, 2.29 (5)°, 81.87 (3)°, 1.18 (4)°, 25.51 (5)°, and 73.68 (4)°, 24.46 (4)°, 81.94 (4)°, 1.11 (4)° and 81.87 (3)° respectively.

There is intermolecular C—H···*Cg*(π) type hydrogen bonds interactions in the crystal structure with the contact distances of 2.9345 Å between acceptor and donor atom and π-ring system defined as C1–C6 ring (Table 1.) and the molecules are stacked along a-axis with these C—H···π type hydrogen-bond interactions (Figure 2.).

### 2. Experimental

A solution of the 2-pyridiylbenzimidazole (1.95 g, 10.0 mmol) in toluene (10 ml) and KOH was added (616 mg, 11.0 mmol) and stirred at 60 °C for 4 h. To this reaction mixture 4-florobenzyl bromide (1.89 g, 10.0 mmol) was added, then heated at this temperature for 24 h. Then volatiles were evaporated in vacuum to dryness. The residue was dissolved in CH_2_Cl_2_ and filtered *via* cannula on celite. The desired product was obtained after concentration of CH_2_Cl_2_ (15 ml) and then precipitating with hexane (30 ml). The off-white solid obtained in 80% yield. *M*.p. 94 °C. ^1^H NMR (400 MHz, CDCl_3_, δ p.p.m.): 6.13 (s, 2H, N—CH_2_); 6.90–6.94 (t, J = 8.0 Hz, 2H, Ar—CH); 7.15–7.18 (m, 2H, Ar—CH); 7.26–7.32 (m, 4H, Ar—CH); 7.81–7.86 (m, 2H, Ar—CH); 8.44 (d, J = 8.0 Hz, ^1^H, Ar—CH); 8.62 (d, J = 4.0 Hz, ^1^H, Ar—CH). ^13^C NMR (100.56 MHz, CDCl_3_, δ p.p.m.): 110.2; 115.1; 120.1; 122.2; 123.8; 124.3; 128.0; 142.2; 148.5; 150.1; 161.4. ^19^F NMR (376.266 MHz, CDCl_3_, δ p.p.m.): - 115.59.

### Crystal data

**Table:**

C_19_H_14_FN_3_	*F*(000) = 632
*M**_r_* = 303.33	*D*_x_ = 1.323 Mg m^−^^3^
Monoclinic, *P*2_1_/*c*	Mo *K*α radiation, λ = 0.71073 Å
Hall symbol: -P 2ybc	Cell parameters from 0 reflections
*a* = 4.7363 (5) Å	θ = 2.4–26.7°
*b* = 15.4102 (17) Å	µ = 0.09 mm^−^^1^
*c* = 20.953 (2) Å	*T* = 296 K
β = 95.363 (8)°	Stick, orange
*V* = 1522.6 (3) Å^3^	0.2 × 0.2 × 0.2 mm
*Z* = 4	

### Data collection

**Table:**

Bruker APEXII CCD diffractometer	3133 independent reflections
Radiation source: sealed tube	2355 reflections with *I* > 2σ(*I*)
Graphite monochromator	*R*_int_ = 0.027
φ and ω scans	θ_max_ = 26.7°, θ_min_ = 2.4°
Absorption correction: multi-scan (Blessing, 1995)	*h* = −5→5
*T*_min_ = 0.984, *T*_max_ = 0.984	*k* = −19→18
13325 measured reflections	*l* = −26→22

### Refinement

**Table:**

Refinement on *F*^2^	Primary atom site location: structure-invariant direct methods
Least-squares matrix: full	Secondary atom site location: difference Fourier map
*R*[*F*^2^ > 2σ(*F*^2^)] = 0.038	Hydrogen site location: mixed
*wR*(*F*^2^) = 0.103	H-atom parameters constrained
*S* = 0.93	*w* = 1/[σ^2^(*F*_o_^2^) + (0.0502*P*)^2^ + 0.1779*P*] where *P* = (*F*_o_^2^ + 2*F*_c_^2^)/3
3133 reflections	(Δ/σ)_max_ = 0.001
208 parameters	Δρ_max_ = 0.14 e Å^−^^3^
1 restraint	Δρ_min_ = −0.17 e Å^−^^3^

### Special details

**Table:**

Geometry. All esds (except the esd in the dihedral angle between two l.s. planes) are estimated using the full covariance matrix. The cell esds are taken into account individually in the estimation of esds in distances, angles and torsion angles; correlations between esds in cell parameters are only used when they are defined by crystal symmetry. An approximate (isotropic) treatment of cell esds is used for estimating esds involving l.s. planes.
Refinement. Refinement of F^2^ against ALL reflections. The weighted R-factor wR and goodness of fit S are based on F^2^, conventional R-factors R are based on F, with F set to zero for negative F^2^. The threshold expression of F^2^ > 2sigma(F^2^) is used only for calculating R-factors(gt) etc. and is not relevant to the choice of reflections for refinement. R-factors based on F^2^ are statistically about twice as large as those based on F, and R- factors based on ALL data will be even larger.

### Fractional atomic coordinates and isotropic or equivalent isotropic displacement parameters (Å^2^)

**Table:**

	*x*	*y*	*z*	*U*_iso_*/*U*_eq_	
N1	0.3612 (2)	0.95634 (7)	0.32306 (5)	0.0598 (3)	
N2	0.1288 (2)	1.03423 (7)	0.24400 (6)	0.0669 (3)	
C14	0.3980 (3)	0.81860 (8)	0.38380 (6)	0.0574 (3)	
C6	0.0419 (3)	1.05982 (8)	0.30241 (7)	0.0629 (3)	
C5	0.1882 (3)	1.01343 (8)	0.35209 (7)	0.0607 (3)	
C13	0.5514 (3)	0.89547 (9)	0.35911 (7)	0.0661 (4)	
H13A	0.6512	0.9256	0.3951	0.079*	
H13B	0.6915	0.8750	0.3317	0.079*	
C7	0.3181 (3)	0.97274 (8)	0.25818 (7)	0.0602 (3)	
C17	0.1266 (4)	0.67652 (9)	0.42898 (8)	0.0785 (4)	
F	−0.0115 (3)	0.60689 (7)	0.45190 (6)	0.1195 (4)	
C1	−0.1590 (3)	1.12201 (9)	0.31618 (9)	0.0762 (4)	
H1	−0.2638	1.1521	0.2836	0.091*	
C4	0.1534 (3)	1.02938 (10)	0.41615 (8)	0.0719 (4)	
H4	0.2555	0.9989	0.4490	0.086*	
C15	0.2114 (3)	0.76997 (10)	0.34407 (7)	0.0736 (4)	
H15	0.1766	0.7856	0.3012	0.088*	
N3	0.5846 (3)	0.85244 (9)	0.22169 (7)	0.0831 (4)	
C19	0.4444 (3)	0.79317 (11)	0.44710 (7)	0.0770 (4)	
H19	0.5696	0.8248	0.4749	0.092*	
C16	0.0746 (4)	0.69844 (11)	0.36643 (8)	0.0856 (5)	
H16	−0.0506	0.6661	0.3391	0.103*	
C8	0.4713 (3)	0.93024 (9)	0.20851 (7)	0.0635 (4)	
C11	0.7609 (4)	0.85310 (15)	0.11857 (10)	0.0983 (6)	
H11	0.8632	0.8251	0.0889	0.118*	
C2	−0.1960 (4)	1.13709 (10)	0.37944 (10)	0.0835 (5)	
H2	−0.3286	1.1781	0.3897	0.100*	
C18	0.3089 (4)	0.72168 (11)	0.47001 (8)	0.0865 (5)	
H18	0.3423	0.7050	0.5127	0.104*	
C3	−0.0396 (4)	1.09246 (11)	0.42858 (9)	0.0818 (5)	
H3	−0.0664	1.1056	0.4709	0.098*	
C9	0.4926 (4)	0.97168 (11)	0.15093 (8)	0.0861 (5)	
H9	0.4076	1.0254	0.1428	0.103*	
C12	0.7260 (4)	0.81593 (13)	0.17637 (10)	0.0989 (6)	
H12	0.8060	0.7615	0.1848	0.119*	
C10	0.6421 (5)	0.93216 (14)	0.10555 (9)	0.1017 (6)	
H10	0.6613	0.9593	0.0665	0.122*	

### Atomic displacement parameters (Å^2^)

**Table:**

	*U*^11^	*U*^22^	*U*^33^	*U*^12^	*U*^13^	*U*^23^
N1	0.0538 (6)	0.0525 (6)	0.0728 (7)	−0.0057 (5)	0.0044 (5)	−0.0023 (5)
N2	0.0652 (7)	0.0542 (6)	0.0812 (8)	−0.0007 (6)	0.0058 (6)	0.0017 (5)
C14	0.0518 (7)	0.0561 (7)	0.0633 (8)	0.0052 (6)	−0.0008 (6)	−0.0061 (6)
C6	0.0578 (7)	0.0471 (7)	0.0847 (9)	−0.0100 (6)	0.0115 (7)	−0.0031 (6)
C5	0.0533 (7)	0.0503 (7)	0.0792 (9)	−0.0144 (6)	0.0099 (6)	−0.0060 (6)
C13	0.0524 (7)	0.0691 (9)	0.0748 (9)	−0.0043 (6)	−0.0048 (6)	−0.0033 (7)
C7	0.0565 (7)	0.0506 (7)	0.0730 (9)	−0.0085 (6)	0.0037 (6)	−0.0018 (6)
C17	0.1041 (12)	0.0522 (8)	0.0826 (10)	0.0003 (8)	0.0259 (9)	−0.0004 (7)
F	0.1701 (11)	0.0738 (6)	0.1201 (9)	−0.0228 (7)	0.0432 (8)	0.0116 (6)
C1	0.0679 (9)	0.0543 (8)	0.1076 (12)	−0.0041 (7)	0.0143 (8)	−0.0013 (8)
C4	0.0684 (9)	0.0682 (9)	0.0801 (10)	−0.0188 (8)	0.0117 (7)	−0.0090 (7)
C15	0.0845 (10)	0.0723 (9)	0.0620 (8)	−0.0157 (8)	−0.0036 (7)	−0.0007 (7)
N3	0.0868 (9)	0.0744 (9)	0.0890 (9)	0.0175 (7)	0.0135 (7)	−0.0045 (7)
C19	0.0821 (10)	0.0777 (10)	0.0679 (9)	0.0005 (8)	−0.0102 (7)	−0.0059 (7)
C16	0.1046 (12)	0.0715 (10)	0.0801 (10)	−0.0271 (9)	0.0046 (9)	−0.0079 (8)
C8	0.0572 (8)	0.0603 (8)	0.0726 (9)	−0.0070 (6)	0.0036 (6)	−0.0066 (7)
C11	0.0899 (12)	0.1126 (15)	0.0951 (13)	0.0017 (12)	0.0224 (10)	−0.0276 (11)
C2	0.0741 (10)	0.0606 (9)	0.1198 (14)	−0.0072 (8)	0.0304 (10)	−0.0160 (9)
C18	0.1154 (13)	0.0768 (11)	0.0666 (9)	0.0084 (10)	0.0046 (9)	0.0092 (8)
C3	0.0811 (11)	0.0713 (10)	0.0966 (12)	−0.0190 (9)	0.0276 (9)	−0.0210 (9)
C9	0.1051 (13)	0.0737 (10)	0.0802 (10)	−0.0003 (9)	0.0134 (9)	0.0007 (8)
C12	0.0997 (13)	0.0955 (13)	0.1023 (13)	0.0277 (11)	0.0142 (11)	−0.0161 (11)
C10	0.1226 (16)	0.1067 (15)	0.0795 (11)	−0.0147 (13)	0.0283 (11)	−0.0091 (10)

### Geometric parameters (Å, º)

**Table:**

N1—C7	1.3790 (17)	C4—H4	0.9300
N1—C5	1.3815 (17)	C15—C16	1.382 (2)
N1—C13	1.4612 (17)	C15—H15	0.9300
N2—C7	1.3192 (17)	N3—C8	1.3319 (19)
N2—C6	1.3845 (18)	N3—C12	1.336 (2)
C14—C15	1.3778 (18)	C19—C18	1.383 (2)
C14—C19	1.381 (2)	C19—H19	0.9300
C14—C13	1.5065 (19)	C16—H16	0.9300
C6—C5	1.393 (2)	C8—C9	1.377 (2)
C6—C1	1.399 (2)	C11—C10	1.359 (3)
C5—C4	1.389 (2)	C11—C12	1.364 (3)
C13—H13A	0.9700	C11—H11	0.9300
C13—H13B	0.9700	C2—C3	1.392 (2)
C7—C8	1.4765 (19)	C2—H2	0.9300
C17—C18	1.353 (2)	C18—H18	0.9300
C17—C16	1.354 (2)	C3—H3	0.9300
C17—F	1.3671 (18)	C9—C10	1.379 (2)
C1—C2	1.373 (2)	C9—H9	0.9300
C1—H1	0.9300	C12—H12	0.9300
C4—C3	1.376 (2)	C10—H10	0.9300
			
C7—N1—C5	106.14 (11)	C16—C15—H15	119.2
C7—N1—C13	130.90 (12)	C8—N3—C12	116.78 (15)
C5—N1—C13	122.93 (12)	C14—C19—C18	121.46 (14)
C7—N2—C6	104.92 (12)	C14—C19—H19	119.3
C15—C14—C19	117.49 (13)	C18—C19—H19	119.3
C15—C14—C13	121.54 (12)	C17—C16—C15	118.58 (15)
C19—C14—C13	120.95 (12)	C17—C16—H16	120.7
N2—C6—C5	110.25 (12)	C15—C16—H16	120.7
N2—C6—C1	129.90 (14)	N3—C8—C9	122.55 (15)
C5—C6—C1	119.85 (14)	N3—C8—C7	117.91 (13)
N1—C5—C4	131.82 (14)	C9—C8—C7	119.54 (14)
N1—C5—C6	105.78 (12)	C10—C11—C12	118.21 (18)
C4—C5—C6	122.37 (14)	C10—C11—H11	120.9
N1—C13—C14	112.85 (10)	C12—C11—H11	120.9
N1—C13—H13A	109.0	C1—C2—C3	121.54 (15)
C14—C13—H13A	109.0	C1—C2—H2	119.2
N1—C13—H13B	109.0	C3—C2—H2	119.2
C14—C13—H13B	109.0	C17—C18—C19	118.58 (15)
H13A—C13—H13B	107.8	C17—C18—H18	120.7
N2—C7—N1	112.87 (12)	C19—C18—H18	120.7
N2—C7—C8	121.89 (13)	C4—C3—C2	121.72 (16)
N1—C7—C8	125.19 (13)	C4—C3—H3	119.1
C18—C17—C16	122.33 (15)	C2—C3—H3	119.1
C18—C17—F	118.58 (15)	C8—C9—C10	118.93 (17)
C16—C17—F	119.09 (16)	C8—C9—H9	120.5
C2—C1—C6	117.76 (16)	C10—C9—H9	120.5
C2—C1—H1	121.1	N3—C12—C11	124.37 (18)
C6—C1—H1	121.1	N3—C12—H12	117.8
C3—C4—C5	116.67 (16)	C11—C12—H12	117.8
C3—C4—H4	121.7	C11—C10—C9	119.15 (18)
C5—C4—H4	121.7	C11—C10—H10	120.4
C14—C15—C16	121.56 (14)	C9—C10—H10	120.4
C14—C15—H15	119.2		
			
C7—N2—C6—C5	1.27 (14)	C19—C14—C15—C16	0.1 (2)
C7—N2—C6—C1	−178.94 (13)	C13—C14—C15—C16	178.76 (15)
C7—N1—C5—C4	−176.42 (14)	C15—C14—C19—C18	−0.1 (2)
C13—N1—C5—C4	1.7 (2)	C13—C14—C19—C18	−178.73 (14)
C7—N1—C5—C6	1.84 (13)	C18—C17—C16—C15	−0.5 (3)
C13—N1—C5—C6	179.92 (11)	F—C17—C16—C15	178.77 (15)
N2—C6—C5—N1	−1.96 (14)	C14—C15—C16—C17	0.2 (3)
C1—C6—C5—N1	178.22 (11)	C12—N3—C8—C9	0.9 (2)
N2—C6—C5—C4	176.50 (12)	C12—N3—C8—C7	−178.95 (14)
C1—C6—C5—C4	−3.32 (19)	N2—C7—C8—N3	−158.51 (13)
C7—N1—C13—C14	−106.39 (15)	N1—C7—C8—N3	24.2 (2)
C5—N1—C13—C14	76.04 (15)	N2—C7—C8—C9	21.6 (2)
C15—C14—C13—N1	50.84 (18)	N1—C7—C8—C9	−155.75 (14)
C19—C14—C13—N1	−130.59 (14)	C6—C1—C2—C3	0.1 (2)
C6—N2—C7—N1	−0.06 (15)	C16—C17—C18—C19	0.6 (3)
C6—N2—C7—C8	−177.69 (11)	F—C17—C18—C19	−178.74 (15)
C5—N1—C7—N2	−1.16 (14)	C14—C19—C18—C17	−0.2 (3)
C13—N1—C7—N2	−179.03 (12)	C5—C4—C3—C2	1.1 (2)
C5—N1—C7—C8	176.39 (12)	C1—C2—C3—C4	−1.9 (2)
C13—N1—C7—C8	−1.5 (2)	N3—C8—C9—C10	−1.4 (3)
N2—C6—C1—C2	−177.38 (14)	C7—C8—C9—C10	178.46 (15)
C5—C6—C1—C2	2.40 (19)	C8—N3—C12—C11	0.1 (3)
N1—C5—C4—C3	179.55 (13)	C10—C11—C12—N3	−0.7 (3)
C6—C5—C4—C3	1.5 (2)	C12—C11—C10—C9	0.2 (3)

### Hydrogen-bond geometry (Å, º)

Cg is the centroid of the C1–C6 ring.

**Table:**

*D*—H···*A*	*D*—H	H···*A*	*D*···*A*	*D*—H···*A*
C13—H13*A*···*Cg*^i^	0.97	2.94	3.486 (2)	117

Symmetry code: (i) *x*−1, *y*, *z*.
